# Hedgehog Signals Mediate Anti-Cancer Drug Resistance in Three-Dimensional Primary Colorectal Cancer Organoid Culture

**DOI:** 10.3390/ijms19041098

**Published:** 2018-04-06

**Authors:** Tatsuya Usui, Masashi Sakurai, Koji Umata, Mohamed Elbadawy, Takashi Ohama, Hideyuki Yamawaki, Shoichi Hazama, Hiroko Takenouchi, Masao Nakajima, Ryouichi Tsunedomi, Nobuaki Suzuki, Hiroaki Nagano, Koichi Sato, Masahiro Kaneda, Kazuaki Sasaki

**Affiliations:** 1 Laboratory of Veterinary Pharmacology, Department of Veterinary Medicine, Faculty of Agriculture, Tokyo University of Agriculture and Technology, 3-5-8 Saiwai-cho, Fuchu, Tokyo 183-8509, Japan; skazuaki@cc.tuat.ac.jp; 2 Laboratory of Veterinary Pathology, Joint Faculty of Veterinary Medicine, Yamaguchi University, 1677-1 Yoshida, Yamaguchi 753-8515, Japan; sakurai@yamaguchi-u.ac.jp; 3 Laboratory of Veterinary Pharmacology, Joint Faculty of Veterinary Medicine, Yamaguchi University, 1677-1 Yoshida, Yamaguchi 753-8515, Japan; t007tb@yamaguchi-u.ac.jp (K.U.); t.ohama@yamaguchi-u.ac.jp (T.O.); k-sato@yamaguchi-u.ac.jp (K.S.); 4 Department of Pharmacology, Faculty of Veterinary Medicine, Benha University, Moshtohor, Toukh, Elqaliobiya 13736, Egypt; mohamed.elbadawy@fvtm.bu.edu.eg; 5 Laboratory of Veterinary Pharmacology, School of Veterinary Medicine, Kitasato University, Higashi 23 bancho 35-1, Towada City, Aomori 034-8628, Japan; yamawaki@vmas.kitasato-u.ac.jp; 6 Department of Translational Research and Developmental Therapeutics against Cancer, School of Medicine, Yamaguchi University, 1-1-1 Ogushi, Ube, Yamaguchi 755-8505, Japan; hazama@yamaguchi-u.ac.jp; 7 Department of Gastroenterological, Breast and Endocrine Surgery, Graduate School of Medicine, Yamaguchi University, 1-1-1 Ogushi, Ube, Yamaguchi 755-8505, Japan; h-take@yamaguchi-u.ac.jp (H.T.); masao-y@yamaguchi-u.ac.jp (M.N.); tsune-r@yamaguchi-u.ac.jp (R.T.); nobusuzu@yamaguchi-u.ac.jp (N.S.); hnagano@yamaguchi-u.ac.jp (H.N.); 8 Laboratory of Veterinary Anatomy, Department of Veterinary Medicine, Faculty of Agriculture, Tokyo University of Agriculture and Technology, 3-5-8 Saiwai-cho, Fuchu, Tokyo 183-8509, Japan; kanedam@cc.tuat.ac.jp

**Keywords:** colorectal cancer, Hedgehog signal, chemoresistance, organoid, stem cell

## Abstract

Colorectal cancer is one of the most common causes of cancer death worldwide. In patients with metastatic colorectal cancer, combination treatment with several anti-cancer drugs is employed and improves overall survival in some patients. Nevertheless, most patients with metastatic disease are not cured owing to the drug resistance. Cancer stem cells are known to regulate resistance to chemotherapy. In the previous study, we established a novel three-dimensional organoid culture model from tumor colorectal tissues of human patients using an air–liquid interface (ALI) method, which contained numerous cancer stem cells and showed resistance to 5-fluorouracil (5-FU) and Irinotecan. Here, we investigate which inhibitor for stem cell-related signal improves the sensitivity for anti-cancer drug treatment in tumor ALI organoids. Treatment with Hedgehog signal inhibitors (AY9944, GANT61) decreases the cell viability of organoids compared with Notch (YO-01027, DAPT) and Wnt (WAV939, Wnt-C59) signal inhibitors. Combination treatment of AY9944 or GANT61 with 5-FU, Irinotecan or Oxaliplatin decreases the cell viability of tumor organoids compared with each anti-cancer drug alone treatment. Treatment with AY9944 or GANT61 inhibits expression of stem cell markers c-Myc, CD44 and Nanog, likely through the decrease of their transcription factor, GLI-1 expression. Combination treatment of AY9944 or GANT61 with 5-FU or Irinotecan also prevents colony formation of colorectal cancer cell lines HCT116 and SW480. These findings suggest that Hedgehog signals mediate anti-cancer drug resistance in colorectal tumor patient-derived ALI organoids and that the inhibitors are useful as a combinational therapeutic strategy against colorectal cancer.

## 1. Introduction

Colorectal cancer is one of the most common causes of cancer-related mortality worldwide [1,2]. Patients are conventionally treated with surgery, chemotherapy and radiotherapy. However, about 50% of patients are diagnosed at late stage and develop liver metastasis, which leads to the low survival rate [3]. In patients with metastatic colorectal cancer, the combination treatment of 5-fluorouracil (5-FU), Folinic acid and Oxaliplatin (FOLFOX) (5-FU), Folinic acid and Irinotecan (FOLFIRI) is employed and improves overall survival in some patents. However, most patients develop a drug resistance during the course of treatment [4]. Therefore, understanding the mechanisms underlying the resistance is essential for the development of effective treatments.

Recent studies have gradually revealed that cancer stem cells are associated with chemoresistance of colorectal cancer [5]. Cancer stem cells exhibit alterations of DNA repair and express ABC membrane transporters mediating chemoresistance [6]. Since many tumors including colorectal cancer might progress due to the cancer stem cells that are not sensitive to the treatment, there is an emerging need for novel therapies targeting cancer stem cells.

Cancer stem cells use various signal pathways such as Wnt, Notch and Hedgehog [6]. Recently, inhibition of Notch signals has been identified as an approach to colorectal cancer [7]. Small molecule antagonists targeting Wnt signals also inhibited proliferation of colon cancer cells [8]. Furthermore, combination treatment with Notch signal inhibitor and Irinotecan reduced tumor growth [9]. These reports imply that inhibition of cancer stem cell-related signals could be useful for the effective treatments through upregulating the sensitivity for anti-cancer drugs. Nevertheless, the detailed mechanisms remain unclear owing to the lack of proper culture model for cancer stem cells from colorectal cancer patients.

In the previous study, we established three-dimensional (3D) organoid model from tumor colorectal tissues of human patients using an air–liquid interface (ALI) method [10]. Tumor ALI organoids consisted of both epithelial and mesenchymal components and closely recapitulated epithelium structures of the original tumor. In addition, they contained numerous cancer stem cells expressing LGR5 and CD44. These characteristics indicate that they could recapitulate tumor microenvironment in the 3D culture. Using this model, we for the first time demonstrated that tumor ALI organoids are more resistant to toxicity of 5-FU and Irinotecan than colorectal cancer cell lines, such as SW480, SW620 and HCT116 [10].

Nevertheless, the resistant mechanisms remain unclear. Here, we investigated whether inhibitors for cancer stem cell-related signal improve the anti-cancer drug resistance of organoids. Among several signal inhibitors, the resistance of tumor ALI organoids is improved by the combination treatment with Hedgehog signal inhibitors and anti-cancer drugs through the decrease of their stemness. The combination treatment also prevents colony formation of colorectal cancer cell lines. 

## 2. Results

### 2.1. Effects of Stem Cell-Related Signal Inhibitors on Cell Viability of Tumor Organoids

**Figure 1:**
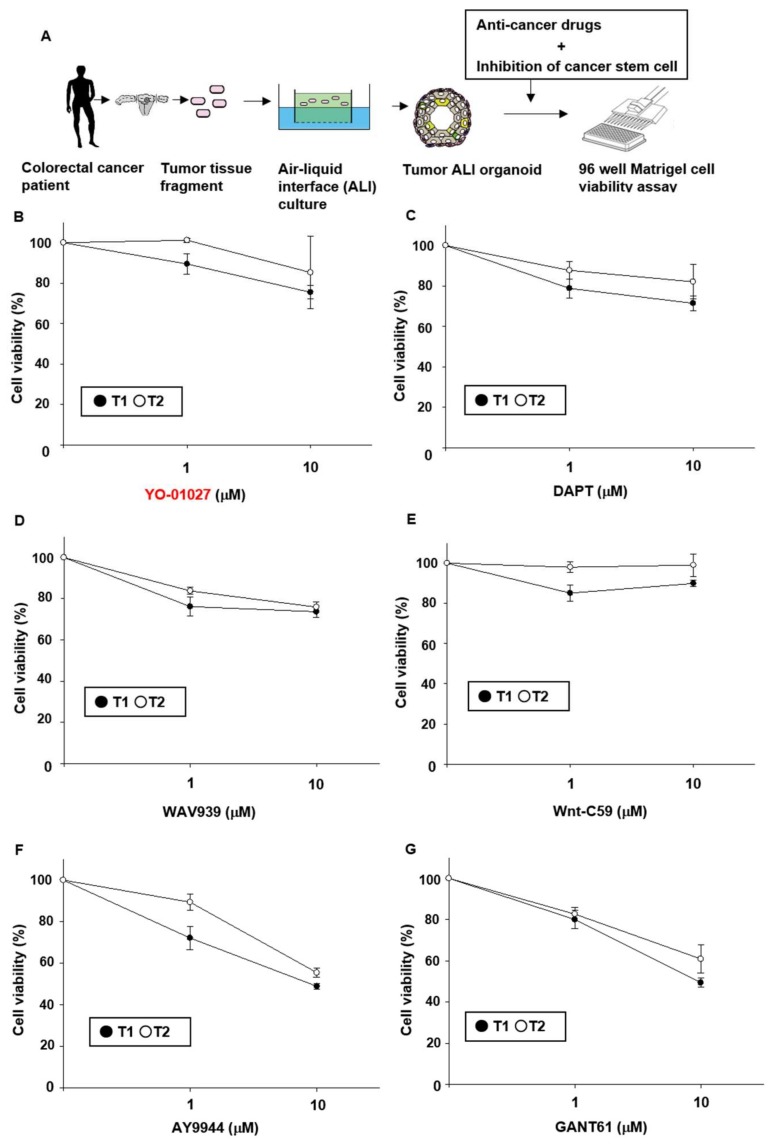
Effects of stem cell-related signal inhibitors on cell viability of tumor air–liquid interface (ALI) organoids. Schematic experimental design of co-treatment with anti-cancer drugs and stem cell-related signal inhibitors in colorectal cancer patient-derived ALI organoids. After tumor organoids were seeded into Matrigel, they were treated with stem cell-related signal inhibitors in the absence or presence of anti-cancer drugs for six days (**A**). Tumor ALI organoids were treated with: Notch signal inhibitors YO-01027 (1–10 μM) (**B**) and DAPT (1–10 μM) (**C**); Wnt signal inhibitors, WAV939 (1–10 μM) (**D**) and Wnt-C59 (1–10 μM) (**E**); or Hedgehog signal inhibitors, AY9944 (1–10 μM) (**F**) and GANT61 (1–10 μM) (**G**) for six days (*n* = 6 each for two patients (T1, T2)). Cell viability was determined using an alamablue assay and 100% represents cell viability of each control.

In the previous study, we established ALI tumor organoids from colorectal cancer patients [10]. Although the tumor ALI organoids showed resistance to 5-FU and Irinotecan, and contained numerous cancer stem cells, the relationship between the resistance and high stemness remains unclear. To explore it, we cultured the tumor ALI organoids with a stem cell-related signal inhibitor in the absence or presence of anti-cancer drugs (Figure 1A). We first examined which stem cell-related signal inhibitors (alone treatment) affect the cell viability of tumor ALI organoids prepared from two patients. Treatment with Notch signal inhibitors, YO-01027 (Figure 1B) and DAPT (Figure 1C), or Wnt signal inhibitors, WAV939 (Figure 1D) and Wnt-C59 (Figure 1E), had minimal effects on the cell viability. On the other hand, treatment with Hedgehog signal inhibitors, AY9944 (Figure 1F) and GANT61 (Figure 1G), decreased the cell viability in a dose-dependent manner. These results imply that activation of Hedgehog signals might play a substantial role on cell viability of tumor organoids compared with other stem cell-related signals.

### 2.2. Effects of Hedgehog Signal Inhibitors on the Sensitivity for Anti-Cancer Drugs in Tumor Organoids

**Figure 2:**
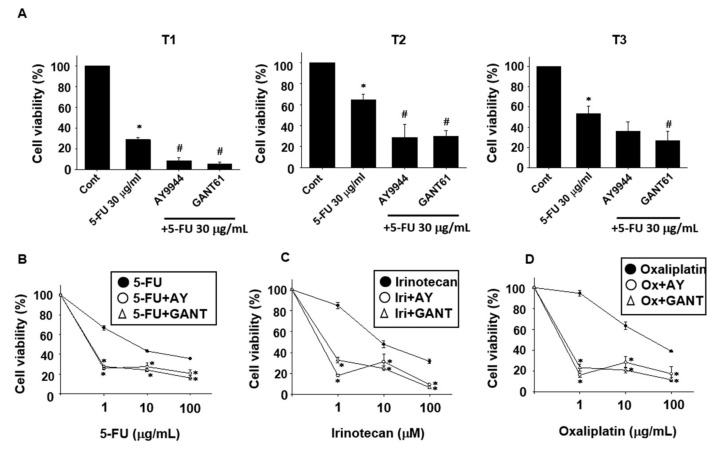
Effects of Hedgehog signal inhibitors on the sensitivity for anti-cancer drugs in tumor ALI organoids. After tumor ALI organoids were seeded into Matrigel, they were treated with 5-FU (30 μg/mL) in the presence or absence of AY9944 or GANT61 for six days (**A**) (*n* = 6 each for three patients (T1, T2, T3)). Cell viability was determined using an alamablue assay and 100% represents cell viability of each control. * *p* < 0.05 vs. Cont. # *p* < 0.05 vs. 5-FU. Effects of Hedgehog signal inhibitors on cell death induced by various types of anti-cancer drugs in tumor organoids. After tumor ALI organoids were seeded into Matrigel, they were treated with: 5-FU (1–100 μg/mL) (**B**); Irinotecan (1–100 μM) (**C**); or Oxaliplatin (1–100 μg/mL) (**D**) in the presence or absence of AY9944 (10 μM) or GANT61 (10 μM) for six days (*n* = 6). Cell viability was determined using an alamablue assay and 100% represents cell viability of each control. * *p* < 0.05 vs. 5-FU (**B**). * *p* < 0.05 vs. Irinotecan (**C**). * *p* < 0.05 vs. Oxaliplatin (**D**).

To examine the additive effects of Hedgehog inhibitors on the cell viability of tumor ALI organoids treated with anti-cancer drugs, we next examined whether co-treatment with Hedgehog signal inhibitors and anti-cancer drugs affects cell viability of tumor organoids. Treatment with 5-FU (30 μg/mL) significantly decreased cell viability of organoids in each patient culture (Figure 2A). Co-treatment with AY9944 or GANT61 significantly decreased the cell viability compared with 5-FU alone treatment in each patient culture (Figure 2A). We further examined the effects of Hedgehog inhibitors on the sensitivity for other anti-cancer drugs at varying concentrations. Co-treatment with AY9944 or GANT61 significantly decreased the cell viability of 5-FU (Figure 2B), Irinotecan (Figure 2C) or Oxaliplatin (Figure 2D) treated organoids at any concentration. These results indicate that activation of Hedgehog signals might be required for anti-cancer drug resistance of tumor ALI organoids. 

### 2.3. Effects of Hedgehog Signal Inhibitors on Expression of Stem Cell Marker Proteins in Tumor Organoids

**Figure 3:**
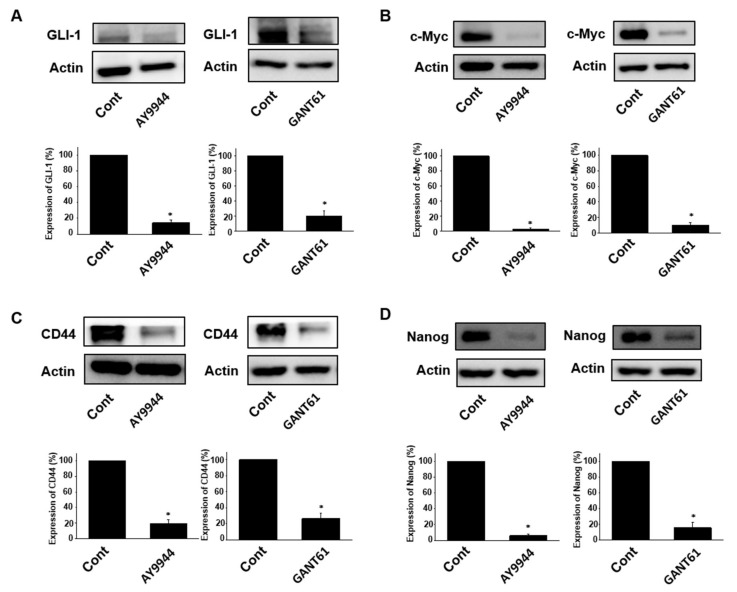
Effects of Hedgehog signal inhibitors on expression of stem cell marker proteins in tumor ALI organoids. After the organoids were treated with GANT61 (10 μM) or AY9944 (10 μM) for six days, protein expression was determined by Western blotting: GLI-1 (*n* = 4–5) (**A**); c-Myc (*n* = 4–5) (**B**); CD44 (*n* = 4–5) (**C**); and Nanog (*n* = 4–5) (**D**). Equal protein loading was confirmed using total actin antibody. * *p* < 0.05 vs. Cont.

Hedgehog signals are activated by binging of Hedgehog ligands to the transmembrane receptor, PTCH1. The bindings release the inhibition of SMO protein. SMO regulates nuclear translocation of GLI-1 that promotes transcription of target genes, such as c-Myc, CD44 and Nanog [11,12]. To investigate the molecular mechanisms underlying the effects of Hedgehog inhibitors, we performed Western blotting. In tumor ALI organoids, AY9944 and GANT61 significantly inhibited GLI-1 protein expression (Figure 3A). AY9944 and GANT61 also significantly inhibited protein expression of c-Myc (Figure 3B), CD44 (Figure 3C) and Nanog (Figure 3D).

### 2.4. Effects of Co-Treatment with Hedgehog Signal Inhibitors and Anti-Cancer Drugs on Colony Formation in Colorectal Cancer Cell Lines

**Figure 4:**
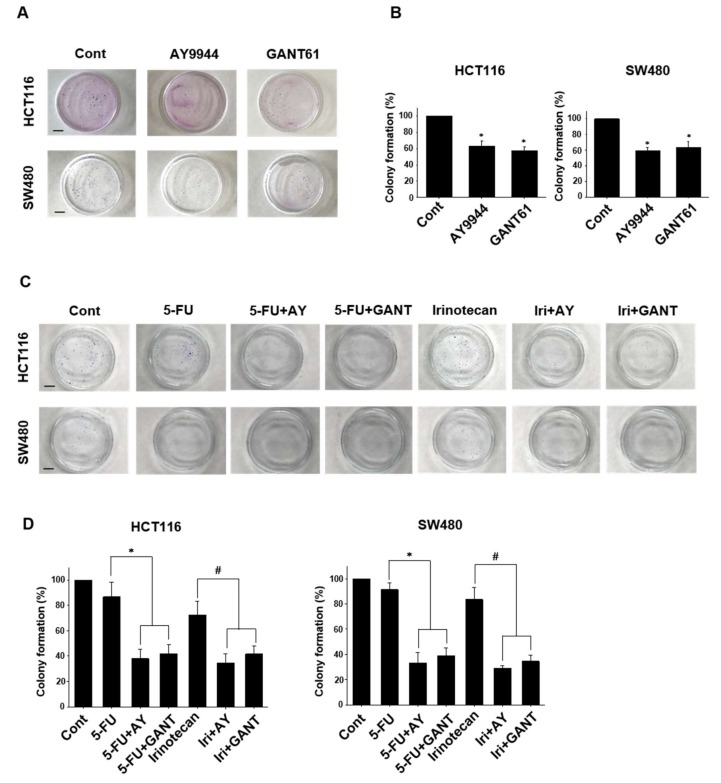
Effects of Hedgehog signal inhibitors on colony formation in colorectal cancer cell lines. After HCT116 and SW-480 cells were seeded on 6 cm-dish, they were treated with GANT61 (5 μM) or AY9944 (5 μM) for six days. Colony formation of HCT116 and SW-480 cells was determined by a colony formation assay. Representative photomicrographs were shown (**A**). Scale bar: 1 cm. After the membranes were fixed with 99.5% ethanol, colonies were stained with Giemsa. The number of colony was counted (*n* = 4) (**B**). * *p* < 0.05 vs. Cont. Effects of co-treatment with Hedgehog signal inhibitors and anti-cancer drugs on colony formation in colorectal cancer cell lines. After HCT116 and SW-480 cells were seeded on 6 cm-dish, they were treated with 5-FU (0.1 μg/mL) or Irinotecan (0.1 μM) in the presence or absence of GANT61 (5 μM) or AY9944 (5 μM) for six days. Representative photomicrographs are shown (**C**). Scale bar: 1 cm. The number of colony was counted (*n* = 6) (**D**). * *p* < 0.05 vs. 5-FU. # *p* < 0.05 vs. Irinotecan.

Since cancer cells with high stemness can colonialize, a colony formation assay is used as an indicator of cancer stemness [13]. Finally, we examined the effects of Hedgehog signal inhibitors on the sensitivity for anti-cancer drugs in colorectal cancer cell lines using a colony formation assay. Treatment with AY9944 or GANT61 significantly decreased the number of colony formation in HCT116 and SW480 (Figure 4A,B). Treatment with low concentration of 5-FU (0.1 μg/mL) or Irinotecan (0.1 μM) had minimal effects on the number of colony formation in HCT116 and SW480. Combination treatment of AY9944 or GANT61 with 5-FU or Irinotecan significantly decreased the number of colony formation in HCT116 and SW480 compared with 5-FU or Irinotecan alone treatment (Figure 4C,D).

## 3. Discussion

**Figure 5:**
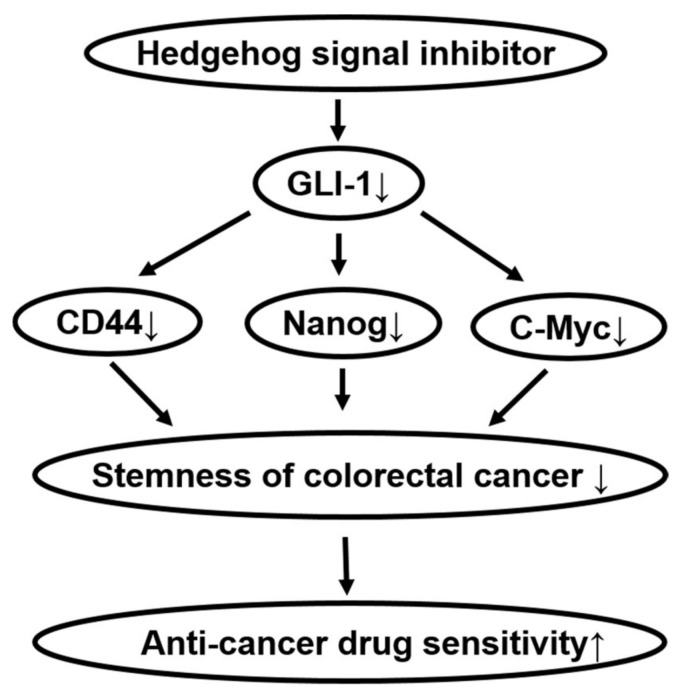
Summary of the present results. GLI-1-targeted Hedgehog signal inhibitors improved the sensitivity for anti-cancer drug treatment in tumor ALI organoids at least in part through the decrease of CD44, Nanog, and c-Myc expression.

The major findings of the present study are as follows: (1) Hedgehog signal inhibitors were more effective on decreasing the cell viability of tumor organoids compared with Wnt and Notch signal inhibitors (Figure 1). (2) The combination treatment with Hedgehog signal inhibitor and 5-FU, Irinotecan or Oxaliplatin effectively decreased the cell viability of tumor organoids (Figure 2). (3) In tumor organoids, expression of c-Myc, CD44 and Nanog was inhibited by Hedgehog signal inhibitors likely through the decrease of GLI-1 expression (Figure 3). (4) The combination treatment with Hedgehog inhibitor and 5-FU or Irinotecan prevented colony formation of HCT116 and SW480 (Figure 4). Collectively, our results indicate that Hedgehog signals regulate anti-cancer drug resistance of human colorectal tumor patient-derived ALI organoids (Figure 5) and that the inhibitors are useful as a combinational therapeutic strategy against colorectal cancer. 

Resistance to anti-cancer drugs in colorectal cancer patients is a major problem. GLI-1 is known to be vital in cancer biology and overexpressed in colorectal cancer cells [14,15,16]. Recent study showed that GLI-1 expression was elevated in 5-FU resistant colorectal cancer cell line LoVo-R compared with non-resistant one [17]. In the same report, it was also shown that knockdown of GLI-1 gene decreased the resistance to 5-FU. Nevertheless, there was no evidence showing that GLI-1 mediates anti-cancer drug resistance in primary colorectal cancer cells. In the present study, we for the first time demonstrated that Hedgehog signal inhibitor decreased the resistance to 5-FU, Irinotecan and Oxaliplatin likely through the inhibition of GLI-1 expression using colorectal cancer patient-derived ALI organoids (Figure 2 and Figure 3). Although the detailed mechanisms by which GLI-1 regulates the resistance remain unclear, further studies by using the ALI organoid system might contribute to overcoming chemoresistance in colorectal cancer patients. 

Tumor microenvironment mediates cancer initiation, progression and metastasis [18]. In pancreatic cancer, Hedgehog signals are activated in the stromal cells rather than epithelial cells, which supports tumorigenesis [19,20]. In pancreatic cancer model mice, treatment with a Hedgehog signal inhibitor, IPI-926 prevented the tumor progression through the depletion of stromal cells [21]. In colorectal cancer model mice, epithelial cells secrete Hedgehog ligands to maintain the stromal phenotype, which is required for adenoma development [22]. These reports suggest that Hedgehog ligands secreted from tumor cells stimulate the stromal cells to secrete tumor growth factors. In the present study, we showed that Hedgehog signals mediate the chemoresistance of tumor ALI organoids (Figure 2). Since our ALI tumor organoid model contains both epithelial and stromal components, it might become a useful tool to clarify the interacting mechanisms between epithelial and mesenchymal cells, which regulate the resistance of anti-cancer drugs in colorectal cancer. 

Cancer stem cells possess the potential of self-renewal, multi-lineage differentiation and tumorigenicity. In the colorectal cancer treatment, cancer stem cells are known to be associated with resistance to chemotherapy and radiotherapy, which causes the recurrence of cancer and promotes metastasis [23,24,25]. Colorectal cancer stem cells express several cell surface markers, such as CD44, CD24, CD133 and CD146 [26]. The relationship between cancer stem cell markers and Hedgehog signaling has been gradually clarified. For examples, expression of cancer stem cell markers correlated with activation of Hedgehog signals in gemcitabine-resistant pancreatic cancer cells [27]. It was also reported that expression level of Hedgehog-related proteins was higher in gastric cancer stem cells expressing both CD44 and CD24 compared with non-expressing ones [28]. In the present study, we showed that Hedgehog inhibitor increased the sensitivity for anti-cancer drugs (Figure 2) and decreased expression of CD44, c-Myc and Nanog likely through the inhibition of GLI-1 in tumor ALI organoids (Figure 3). These results indicate that combination therapy of Hedgehog inhibitor with anti-cancer drugs might become a promising strategy to remove colorectal cancer stem cells from the patients. 

Survival rate in metastatic colorectal cancer patients has been improved due to major advances in chemotherapy and targeted drugs. Since several studies showed that dysregulation of Hedgehog signals mediates colorectal cancer progression and metastasis [29], Hedgehog signals are regarded as a new therapeutic target for the treatment of colorectal cancer. Hedgehog signal-related protein, SMO regulates nuclear translocation of GLI-1 that promotes transcription of target genes, such as c-Myc and CD44 [11]. Inhibition of SMO has been studied in a variety of tumor types [30]. However, a SMO inhibitor, Vismodegib, did not extend progression-free survival in colorectal cancer patients [31,32]. In addition, acquired resistance to SMO inhibitors also occurred in the clinical test phase [33]. To overcome these problems, more specific and effective inhibitor for Hedgehog signals is required. GANT61 is a small molecule that inhibits binding of GLI-1 and induces DNA double strand breaks [34]. In the present study, we showed that a SMO inhibitor, AY9944 and a GLI-1 inhibitor, GANT61 improved the sensitivity for anti-cancer drugs in tumor ALI organoids (Figure 3). We also showed that combination of AY9944 or GANT61 with 5-FU or Irinotecan prevented the colony formation in SW480 and HCT116 cells (Figure 4). These results indicate that combination of not only SMO inhibitor but also selective GLI-1 inhibitor with anti-cancer drugs might be useful for an effective combinational therapy of colorectal cancer. 

In summary, we for the first time demonstrated that Hedgehog signals mediate the resistance to anti-cancer drugs in human colorectal tissue-derived ALI organoids through the decrease of their stemness. It was also suggested that combinational therapy of Hedgehog inhibitor with anti-cancer drugs is effective for colony formation of colorectal cancer cell lines. Further studies on the relationship between Hedgehog signal and chemotherapy contribute to developing new strategy for colorectal cancer treatment.

## 4. Materials and Methods

### 4.1. Materials 

Human colorectal tumor tissue-derived ALI organoids were cultured as described previously [10]. The medium components were as follows: Advanced Dulbecco’s Modified Eagle’s Medium (DMEM) with 50% Wnt, Noggin and R-Spondin conditioned medium; GlutaMax; B-27 supplement; 100 μg/mL Primocin (Invitrogen, Carlsbad, CA, USA); 1 mM *N*-Acetyl-l-cysteine; 10 mM Nicotinamide (Sigma-Aldrich, St. Louis, MO, USA); 50 ng/mL mouse epidermal growth factor (EGF) (PeproTech, Inc., Rocky Hill, NJ, USA); 500 nM A83-01 (Adooq Bioscience, Irvine, CA, USA); and 3 μM SB202190; 10 μM Y-27632 (Cayman, Ann Arbor, MI, USA). Stem cell-related signal inhibitors were as follows: YO-01027 (Toronto Research Chemicals, Toronto, Canada); DAPT (Adooq Bioscience); WAV939; Wnt-C59; AY9944; and GANT61 (Cayman). Anti-cancer drugs were as follows: 5-FU (WAKO, Tokyo, Japan); Irinotecan (LC Laboratories, Woburn, MA, USA); and Oxaliplatin (Adooq Bioscience). Antibody sources were as follows: GLI-1 (Gene Tex, Irvine, CA, USA); CD44 (Bethyl Laboratories, Montgomery, TX, USA); c-Myc; and Nanog (Cell Signaling, Beverly, MA, USA). Secondary antibodies were as follows: Horseradish peroxidase (HRP) conjugated anti-rabbit IgG; HRP conjugated anti-goat IgG (Cayman); and HRP conjugated anti-mouse IgG (Millipore, Temecula, CA, USA).

### 4.2. Cell Culture 

Colorectal cancer cell lines HCT116 and SW480 were cultured in DMEM supplemented with 10% fetal bovine serum (FBS, Invitrogen, Carlsbad, CA, USA).

### 4.3. Colorectal Tumor Tissues

Tumor samples were obtained from patients who underwent surgery at the Department of Gastroenterological, Breast and Endocrine Surgery, Yamaguchi University Graduate School of Medicine (Yamaguchi, Japan). All patients were diagnosed with colorectal cancer. Pre-operative therapy was never given to any of these patients. From the resected colon segment, tumor tissues were isolated. The isolated tissues were used for the organoid culture. Eight tumor samples were attempted to produce organoids. Among them, we showed the data of five samples in this study. The study protocol was approved by the Institutional Review Board (IRB) for Human Use at Yamaguchi University Hospital. Written informed consent for this study was obtained from all patients prior to surgery (IRB approved numbers are H17-82 and H26-44).

### 4.4. Generation of Three-Dimensional ALI Tumor Colorectal Organoids

Tumor tissues from patients were washed in cold HEPES buffered saline. After the tissues were minced on ice, they were embedded in a collagen gel and cultured in the media using an ALI culture system as previously described [10,35] (Figure 1A). Organoids were passaged every 7–14 days by using a 2000 unit/mL collagenase IV (Worthington, Lakewood, NJ, USA) as described previously [35] [36] and replated into new ALI collagen gels at 1:2–4 split. 

### 4.5. Cell Viability Assay of Organoids

Cell viability assay of organoid was performed as described previously [10]. To evaluate the effects of stem cell inhibitors on organoids, supplementary components containing advanced DMEM (without Wnt, EGF, Noggin and R-Spondin) was used during the assay. After the organoids were trypsinized for 5 min and filtered using a 100 μm cell strainer (Falcon, Cary, NC, USA), 5 × 10^3^ cells of organoids were seeded into 10 μL of Matrigel on a 96 well culture plate and incubated for 24 h. They were then treated with stem cell-related signal inhibitors (YO-01027, DAPT, WAV939, Wnt-C59, AY9944 and GANT61) or anti-cancer drugs (5-FU, Irinotecan and Oxaliplatin) at the varying concentrations for six days. Each cell viability was examined by cell counting using an alamablue kit (Invitrogen). The fluorescence (emission wavelength; 585 nm) was read in a standard plate reader (Beckman Coulter Inc., Irvine, CA, USA).

### 4.6. Western Blotting 

Western blotting was performed as described previously [37,38]. Protein lysates were obtained by homogenizing the cells with Triton-based lysis buffer (50 mM Tris-HCl (pH 8.0), 5 mM EDTA, 5 mM EGTA, 1% Triton X100, 1 mM sodium orthovanadate, 20 mM sodium pyrophosphate, and Roche Complete protease inhibitor mixture). Loading proteins (10–20 μg) were separated by SDS-PAGE (10%) and transferred to a nitrocellulose membrane (Wako, Osaka, Japan). After blocked with 0.5% skim milk, the membranes were incubated with primary antibody (GLI-1; 1:200, c-Myc; 1:500, CD44; 1:200, Nanog; 1:500, total actin; 1:500) at 4 °C overnight. The membranes were incubated with secondary antibody (1:10,000 dilution, 1 h) and ECL Pro (PerkinElmer, Freiburg, Germany). The results were visualized using LAS-3000 (Fujifilm, Tokyo, Japan) and quantified using ImageJ densitometry analysis software (National Institutes of Health, Bethesda, MD, USA).

### 4.7. Colony Formation Assay

Colony formation assay was performed as described previously [39]. HCT116 cells at 5 × 10^2^ or SW480 cells at 1 × 10^3^ were seeded on 60 mm dish and cultured. Next day, the cells were treated with AY9944 or GANT61 in the absence or presence of 5-FU or Irinotecan for six days. After the cells were fixed with 99.5% ethanol, colonies were stained with Giemsa. The number of surviving colonies was counted. 

### 4.8. Statistical Analysis

Data are shown as means ± SEM. Statistical evaluations were performed by Student’s *t*-test between two groups. Groups of more than three were evaluated by one-way ANOVA followed by Bonferroni’s test. Values of *p* < 0.05 were considered statistically significant.
